# Should Sea-Ice Modeling Tools Designed for Climate Research Be Used for Short-Term Forecasting?

**DOI:** 10.1007/s40641-020-00162-y

**Published:** 2020-09-26

**Authors:** Elizabeth Hunke, Richard Allard, Philippe Blain, Ed Blockley, Daniel Feltham, Thierry Fichefet, Gilles Garric, Robert Grumbine, Jean-François Lemieux, Till Rasmussen, Mads Ribergaard, Andrew Roberts, Axel Schweiger, Steffen Tietsche, Bruno Tremblay, Martin Vancoppenolle, Jinlun Zhang

**Affiliations:** 1 MS-B216, Los Alamos National Laboratory, Los Alamos, NM 87545 USA; 2 U.S. Naval Research Laboratory Stennis Space Center, Stennis, MS USA; 3 Centre de prévision météorologique et environnementale du Canada, Environnement et Changement Climatique Canada, Dorval, QC Canada; 4 Met Office Hadley Centre, Exeter, UK; 5 CPOM, University of Reading, Reading, UK; 6 Earth and Life Institute, Université catholique de Louvain, Louvain, Belgium; 7 Mercator Ocean International, Ramonville Saint Agne, France; 8 National Weather Service, National Oceanic and Atmospheric Administration, College Park, MD USA; 9 Danish Meteorological Institute, Copenhagen, Denmark; 10 Polar Science Center, University of Washington, Seattle, WA USA; 11 European Centre for Medium-Range Weather Forecasts, Reading, UK; 12 McGill University, Montréal, Canada; 13 Laboratoire d’Océanographie et du Climat, CNRS/IRD/MNHN, Sorbonne Université, Paris, France

**Keywords:** Sea ice, Climate, Model, Numerical weather prediction

## Abstract

In theory, the same sea-ice models could be used for both research and operations, but in practice, differences in scientific and software requirements and computational and human resources complicate the matter. Although sea-ice modeling tools developed for climate studies and other research applications produce output of interest to operational forecast users, such as ice motion, convergence, and internal ice pressure, the relevant spatial and temporal scales may not be sufficiently resolved. For instance, sea-ice research codes are typically run with horizontal resolution of more than 3 km, while mariners need information on scales less than 300 m. Certain sea-ice processes and coupled feedbacks that are critical to simulating the Earth system may not be relevant on these scales; and therefore, the most important model upgrades for improving sea-ice predictions might be made in the atmosphere and ocean components of coupled models or in their coupling mechanisms, rather than in the sea-ice model itself. This paper discusses some of the challenges in applying sea-ice modeling tools developed for research purposes for operational forecasting on short time scales, and highlights promising new directions in sea-ice modeling.

## Introduction

Broadly speaking, there are three overlapping communities of sea-ice modelers: those who are interested in understanding and accurately simulating the detailed physical processes, climate modelers who are interested in computationally efficient representations of large-scale sea-ice characteristics and processes, and the operational forecasting community, who are interested in models that produce efficient, skillful predictions for a range of spatio-temporal scales. A fourth community does not perform the modeling work itself but uses the results: policy makers and other stakeholders, such as mariners.

Numerical weather forecasting centers traditionally have used relatively simple sea-ice models^1^, mainly as surface boundary conditions for their atmospheric simulations. However, as codes in the sea-ice and climate research community have matured, and as resources have grown to allow more computationally ambitious simulation procedures and fully coupled atmosphere-ocean-ice configurations, operational centers are increasingly turning toward established sea-ice research codes for numerical forecasting systems. Motivated by recent workshop discussions exploring next-generation sea-ice modeling [1], this paper questions whether forecasting systems should use sea-ice models developed for climate applications.

While they share a common subject, research and operational communities have different goals, requirements, and needs. For the research community (process level through climate scales), understanding and properly representing the physical processes are paramount; forecasters need products that are both skillful and valuable to stakeholders, particularly with respect to hazards and risk [3]. For instance, conservation of mass and heat is crucial for long-term simulations of global change under imposed forcing such as greenhouse gas emissions, in order to detect and distinguish the emergent climate response. For shorter term forecasts, an initial condition is usually imposed by assimilating observed data products, which often precludes conservation. In this paper, we use “short term” to refer to time scales up to seasonal and “long term” or “climate scale” to refer to interannual to centennial time scales.

Sea ice occurs in many forms that present different types of hazards, from solid, stationary shelves of landfast ice to large, brittle plates, to loose mixtures of smaller floes with slushy brash ice and icebergs. Critical elements of a physics-based sea-ice model framework include necessary, first-order physical representations such as basic thermodynamics and dynamics with sound subgrid-scale parameterizations of processes essential to major feedback cycles, relevant coupling mechanisms with the atmosphere and ocean, system memory (e.g., ice volume), awareness of sea ice’s multiscale character, and, for longer time scales, suitably conserved properties such as heat, momentum, and mass of water and salt. As described below, first-order physical processes are currently represented in large-scale Earth system models, but whether they are necessary or sufficient now or in the future depends on the application. For instance, mariners say that area fractions of multiple ice thicknesses or floe sizes are not especially useful for navigation, while locations of high sea-ice pressure and ridged ice are [4]. Climate models, on the other hand, require the ice thickness distribution, which describes the fractional area coverage within each grid cell of ice in a given thickness range, to capture important climate feedback processes [5].

Since the inaugural voyage of the icebreaker Yermak in 1899, mathematical models of sea ice were conceived to serve a dual purpose of short-term prediction and climatic signal detection. In [6], early connections were drawn between sea ice forecasting methods for seasonal Arctic Ocean navigability and global climate warming. Subsequent Russian and American models developed in the 1960s and 1970s used numerical methods for synoptic to seasonal integrations [7–9]. However, the culmination of that research in comprehensive dynamic-thermodynamic sea ice models (e.g., [10]) proved too computationally expensive for coupled climate system integrations. Consequently, simplifications first used for short-term integrations more than a decade earlier, especially to internal sea ice stress, were retrospectively applied in climate investigations (e.g., [11]). More recently, alternative approaches for sea ice dynamics have been developed specifically to improve operational forecasts (e.g., [12]), which could also be useful for climate-scale simulations.

Modelers must balance the physics that can be included in sea ice component models against their computational expense. Internal variability makes the atmosphere difficult to predict [13], and modelers run ensembles of perturbed simulations to capture the “envelope” of potential outcomes, which serves as a measure of uncertainty around a mean response; higher resolution simulations usually impose a reduction in the number of ensemble members that can be run, and on the complexity of the sea ice model.

Another practical consideration is availability and reliability of modeling technologies. Well-vetted, community-developed modeling tools offer attractive options for operational forecasting centers when upgrading their models. While sea-ice processes that are primarily restricted to action in a vertical column are easy to share, dynamical cores are fundamentally linked with the underlying code structure (e.g., meshing considerations associated with Eulerian or Lagrangian approaches) and therefore are more difficult to share. Fortunately, much of sea-ice physics is already represented within column descriptions, and therefore our recommendations focus on dynamics and accelerating community-wide progress through shared model frameworks.

## Modeling Systems

Several models designed for large-scale or long-term (climate) simulation are used in operational forecasting settings to simulate the growth, melting, and movement of sea ice, such as the CICE model [14], the Louvain-la-Neuve Ice Model (LIM, [15]) (now evolving into the Sea Ice Modelling Integrated Initiative (SI^3^, [16]), the Sea Ice Simulator (SIS, [17]), and TED, a thickness-and-enthalpy-distribution sea-ice model [18]. They capture the basic sea ice physics, including variable ice concentration [10, 19], thermodynamics with varying complexity in the representation of salinity (e.g., [15, 20–23]), and a dynamical formulation with a viscous-plastic–based sea-ice rheology, such as elastic-viscous-plastic (EVP [24–28]), elastic-anisotropic-plastic (EAP [29]), and implicit viscous-plastic ice dynamics solvers (e.g., [10, 30]).

Forecasting centers that include sea ice as a component of their modeling systems are shown in Table 1. The ocean-ice resolution in these models is moving toward finer scales, with 1/12^∘^ global models already in use and even higher resolution in regional configurations.

**Table 1:** Selected modeling systems that include sea ice

Country	Institute	Modeling	Ocean	Sea ice	Atmosphere	Ocean/ice	Assimilation
		system	model	model	model	resolution	system
Australia/ USA	BoM/ NCAR	AMPS	Data	Polar mods	Polar WRF	1.67 km	3DVAR
Canada	CCMEP	CAPS	NEMO	CICE	GEM^‡^	0.08^∘^	SAM
Canada	CCMEP	GIOPS	NEMO	CICE	GEM^‡^	0.25^∘^	SAM
Canada	CCMEP	RIOPS	NEMO	CICE	GEM	0.25^∘^	SAM
China	NMEFC	ArcIOPS	MITgcm	MITgcm	GFS	18 km	EnKF
Denmark	DMI	HYCOM-CICE	HYCOM	CICE	IFS^‡^	10 km	nudging
Europe	ECMWF	ECMWF	NEMO	LIM2	IFS	0.25^∘^	NEMOVAR
Europe	UK Met Office	GLO-CPL/CMEMS	NEMO	CICE	UM	0.25^∘^	CPLDA
Finland	FMI	ALADIN-HIRLAM^†^	HBM	HELMI	HarmonEPS	1 n.mi.	3DVAR
Finland	FMI	ALADIN-HIRLAM	HBM	HELMI	HIRLAM	1 n.mi.	4DVAR
France	MOI	GLO-HR/CMEMS	NEMO	LIM2	IFS^‡^	0.08^∘^	SAM
Japan	JMA/MRI	CPS2	MRI.COM	MRI.COM	GSM	0.5^∘^	MOVE
Norway	NERSC / Met Norway	TOPAZ4	HYCOM	TOPAZ	IFS^‡^	12–16 km (NP)	EnKF
UK	Met Office	FOAM	NEMO	CICE	UM^‡^	0.25^∘^	NEMOVAR
UK	Met Office	GloSea	NEMO	CICE	UM	0.25^∘^	NEMOVAR^‡^
USA	NWS	RTOFS	HYCOM	CICE	GFS	3.5 km (NP)	NCODA-based
USA	NWS	CFS	MOM4	SIS1	GFS	0.5^∘^	GODAS
USA	USN	GOFS	HYCOM	CICE	NAVGEM	3.5 km (NP)	NCODA

Acronyms are defined in Table 2. ^‡^Model is run offline. ^†^Variants of this system are used by other members of the HIRLAM Consortium: Denmark, Estonia, Finland, Iceland, Ireland, Netherlands, Norway, Spain, Sweden, Lithuania

**Table 2:** Acronyms

3DVAR	3-D variational analysis method
AMPS	Antarctic Mesoscale Prediction System [86]
ArcIOPS	Arctic Ice Ocean Prediction System [31]
BoM	Australian Bureau of Meteorology
CAPS	Canadian Arctic Prediction System
CCMEP	Canadian Centre for Meteorological and Environmental Prediction
CICE	The Los Alamos Sea Ice Model [14, 87]
CFS	Climate Forecast System [88]
CMEMS	Copernicus Marine Environment Monitoring Service
CPLDA	Coupled atmosphere–land–ocean–ice Data Assimilation system [89]
CPS2	Coupled Prediction System [90]
DMI	Danish Meteorological Institute [91]
ECMWF	European Centre for Medium-Range Weather Forecasts [92]
EAP	elastic-anisotropic-plastic rheology
EnKF	Ensemble Kalman Filter
ESMF	Earth System Modeling Framework [93]
EVP	elastic-viscous-plastic rheology
FMI	Finnish Meteorological Institute
FOAM	Forecasting Ocean Assimilation Model [94]
GEM	Global Environmental Multiscale model [95]
GFS	Global Forecast System [96]
GIOPS	Global Ice Ocean Prediction System [97]
GLO-CPL	Global Coupled System
GLO-HR	Global High Resolution System [98]
GloSea	Global Seasonal forecasting system [99]
GODAS	Global Ocean Data Assimilation System [100]
GOFS	Global Ocean Forecasting System [101, 102]
GSM	Global Spectral Model [103]
HarmonEPS	HIRLAM–ALADIN Research on Mesoscale Operational Numerical weather prediction
	in Euromed (HARMONIE) Ensemble Prediction System [104, 105]
HBM	High-Resolution Operational Model for the Baltic (HIROMB) Baltic Operational
	Oceanographic System (BOOS) Model [106]
HELMI	Helsinki Multi-category sea-Ice model [107]
HIRLAM	High Resolution Limited Area Model [108]
HYCOM	Hybrid Coordinate Ocean Model [109, 110]
IFS	Integrated Forecasting System [92]
JMA/MRI	Japan Meteorological Agency/Meteorological Research Institute
LIM2	Louvain-la-Neuve Ice Model version 2 [111]
LKF	Linear kinematic features
MITgcm	Massachusetts Institute of Technology Global Circulation Model [112]
MIZ	Marginal Ice Zone
MOI	Mercator Ocean International
MOSAiC	Multidisciplinary Drifting Observatory for the Study of Arctic Climate
MOVE	Multivariate Ocean Variational Estimation [113]
MRI.COM	Japanese Meteorological Research Institute Community Ocean Model [114]
NAVGEM	U. S. NAVy Global Environmental Modeling system [115]
NCAR	U. S. National Center for Atmospheric Research
NCODA	U. S. Navy Coupled Ocean Data Assimilation system [116]
NEMO	Nucleus for European Modelling of the Ocean [16]
NEMOVAR	3DVAR data assimilation system for use with NEMO [94, 117]
NERSC	Nansen Environmental and Remote Sensing Center
NMEFC	Chinese National Marine Environmental Forecasting Center
NP	North Pole
NWS	U. S. National Weather Service
Polar WRF	Polar Weather Research and Forecasting model [118]
RIOPS	Regional Ice Ocean Prediction System
RTOFS	Real Time Ocean Forecast System [119]
SAM	Systéme d’Assimilation Mercator [120]
SI^3^	Sea Ice modelling Integrated Initiative [121]
SIS	Sea Ice Simulator [17]
TED	Thickness and Enthalpy Distribution sea-ice model [18]
TOPAZ	The Operational Prediction system for the North Atlantic European coastal Zones [122]
UK	United Kingdom
UM	Unified Model
USA	United States of America
USN	U. S. Navy
VP	viscous-plastic rheology

Data assimilation codes at operational centers are often more complex than, and heavily intertwined with, the physical models. Most of these modeling systems assimilate satellite-derived sea ice concentration, among other ocean properties such as ocean temperature and salinity. Only the ArcIOPS modeling system assimilates ice thickness in an operational mode [31], although approaches using this and other sea ice variables such as ice surface temperature are being explored (e.g., [32–35]).

## Modeling Considerations

Most current, physics-based sea-ice models designed for climate study are expressed in terms of local balances of conserved quantities such as mass, heat, and momentum, and include many parameterizations of unresolved, small-scale processes. Sea-ice physics may be divided into thermodynamic and dynamic processes, which are mostly vertical and horizontal, respectively. There are different models and approaches available for each of these processes, which may be used in different combinations depending on the problem being studied. In this section, we discuss considerations relevant to operational modeling, including scales, processes, data assimilation, and model complexity.

### Scales and Resolution

Relevant temporal and spatial scales for sea-ice information depend on the application. Elements of the ice pack that are relevant for tactical navigation include floes, leads, and ridges, with scales of 10 m to a few kilometers for hours up to a few days; navigation benefits most from information at 300-m resolution or higher [4]. Route planning requires ice information at larger scales, such as ice thickness, drift, and convergence/compression on time scales of days to months. Development of standards and regulations, which requires an understanding of long-term trends, uses climate-scale information more directly. A challenge for Earth system model predictions is the transition from shorter time scales largely controlled by “memory” of initial conditions to longer time scales at which the model’s response to external forcing emerges (decadal and beyond).

Spatial resolution depends on model configuration, ranging from a few kilometers for highly focused, horizontal areas to tens of kilometers for global simulations. In all cases, subgrid-scale processes must be represented via parameterizations. For instance, most models represent an ice thickness distribution, and floe size distributions are being tested in research codes. Vertical resolution ranges from fractions of a centimeter to meters for the sea-ice and snow column.

### Processes

Mariners (e.g., [4]) desire risk analyses and predictions for ice drift and compression, pressure ridges, thickness, and probabilistic information such as comparison of the current ice situation to normal. Ice melting stage, snow cover, and the presence of landfast ice and icebergs are also useful. Model parameterizations already represent much of this information in some form but require careful interpretation of model output. As [3] note, advances in Arctic sea-ice predictability (e.g., [36]) and prediction (e.g., [37]) are not widely utilized for planning and risk mitigation because the native model output variables are not useful, and more useful variables lack reliable skill estimates.

Among the most uncertain representations in sea-ice models are their coupled interactions with the atmosphere and ocean boundary layers. For example, wind forcing is poorly represented at lower resolution, while parameterizations such as drag may break down in the range of floe- and finer scales, complicating the representation of ice and boundary layer coupled processes.

#### Column Physics

Because sea ice is very thin compared with its horizontal extent, and because of the steep thermal gradient for much of the year between the upper (atmospheric) and lower (oceanic) ice surfaces, many thermodynamic processes can be simply described in the vertical direction, to first order. These include surface fluxes (radiation, heat, water, salt, and other bio-chemical constituents), conduction, melting at the top and bottom of the ice column, seawater freezing, brine dynamics within the ice column, and snow-ice formation, in which snow on top of the ice is converted to sea ice through flooding and freezing of sea water.

A primary goal of large-scale sea-ice models (e.g., those used to study climate) is to describe the ice thickness distribution. Each thickness category is usually represented as a single ice column with snow on top, and a full vertical thermodynamic calculation is performed for each, which may also include halodynamics and ecosystem cycling. In addition to thermodynamic growth and melt, ice may be transferred from one thickness category to another through mechanical deformation processes, also known generically as “ridging.” Although ridging is a dynamical process arising from ice convergence and shear, it is wholly described as part of the column physics of current sea-ice models. Ridges are formidable barriers to ship navigation.

Ice aging and melt stage are useful diagnostics for vessels operating in the ice because they indicate the degree of deterioration and strength of the ice. Likewise, snow cover can be indicative of sturdy ice and also creates friction for passing ships. Current models are capable of producing ice age, melt stage, and snow cover, but these diagnostics have generally not been developed and evaluated for operational products.

A new capability now becoming available in large-scale sea-ice models is the floe size distribution (FSD [38–41]). At global scales, the FSD affects rates of change for sea ice in wave-influenced ice areas [42]. Although floe size itself is of less interest to mariners [4], if coupled with melt stage, it could be considered for operational deployment as an indicator of ice damage and/or strength.

#### Dynamics

The dynamics components of sea-ice models capture the spatial character and evolution of the ice pack through processes that affect its momentum, strength, and deformation, and are thus directly relevant to operational forecasting of ice drift, compression, and ridging. The computed velocity is used to conservatively transport the ice horizontally, and divergence and convergence create areas of open water and closely packed, often ridged ice, which affect the large-scale sea-ice state. The ice pack can generally be divided into two regimes: the consolidated pack, in which ice is composed of large, brittle plates that behave as a plastic material, and the marginal ice zone (MIZ), in which ice floes tend to be smaller and less concentrated. The transition region between these two regimes is not well defined and changes in both time and space as the ice pack freezes, melts, and moves around. Vessels operating in the ice pack encounter less resistance in the MIZ than in the consolidated pack, although it is still a very hazardous area; the consolidated pack generally requires icebreaking capabilities, and identifying paths of weaker or less consolidated ice is helpful for transiting the pack. In the Arctic, the MIZ has traditionally been a relatively narrow region around the edges of the consolidated ice pack, but this distinction is changing as the Arctic pack becomes more seasonal and similar to the Antarctic [43].

**Fig. 1:**
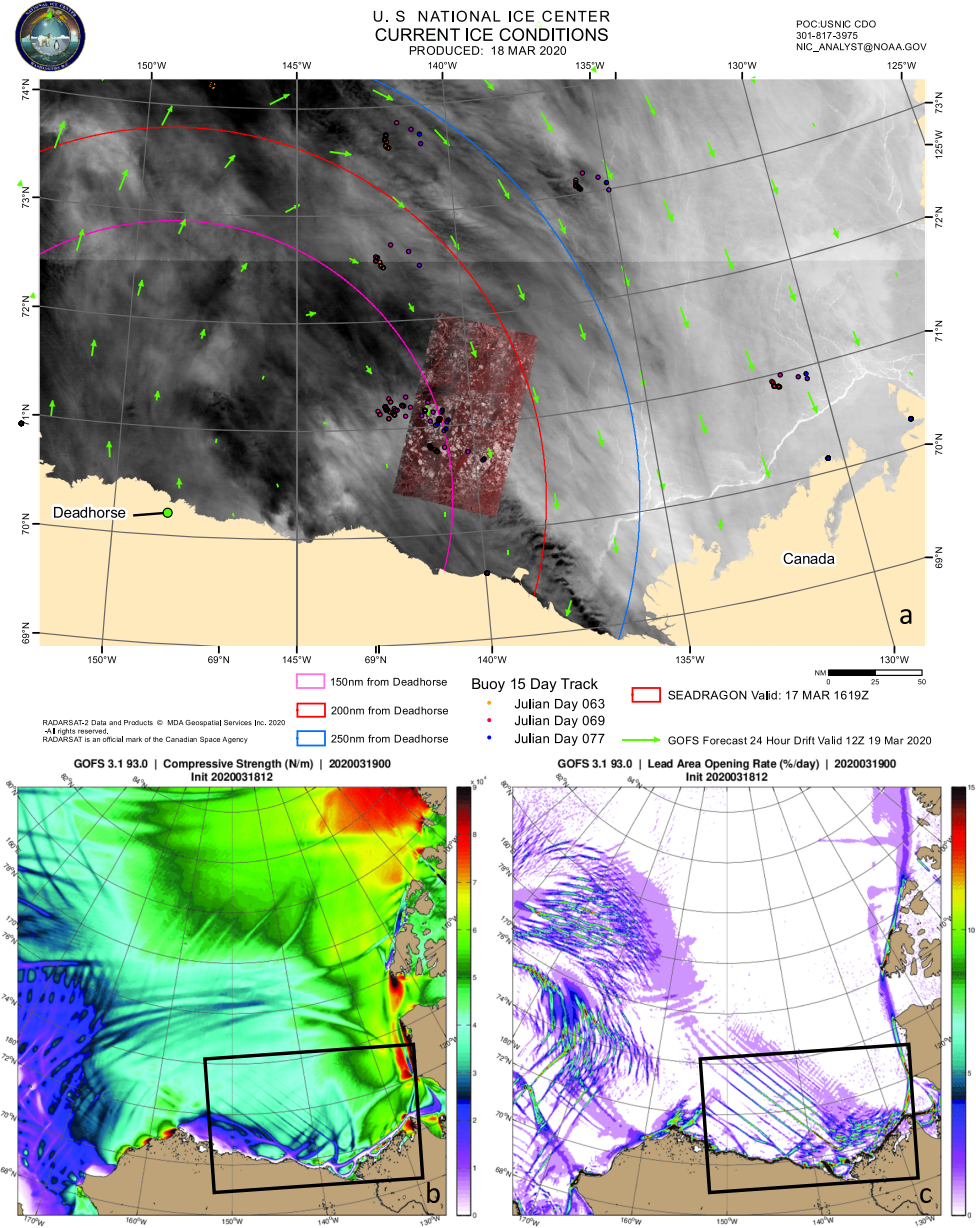
**a** Sea-ice drift forecast from GOFS 3.1 (green vectors) in support of the ICEX joint exercise “Camp Seadragon” in March 2020, overlain on VIIRS and RADARSAT2 (red rectangle) sea-ice imagery [49]. Colored dots show buoys rotating in inertial motion. **b** GOFS sea-ice compressive strength (10^4^ N/m). The ice is weak where it is moving away from the shore and along the shore lead, visible as a white line in the satellite image in **a**. The ice has slowed under compressive conditions in the eastern area of the NIC domain (black box). **c** Sea-ice opening rates (%/day) associated with divergence and shear. Linear kinematic features appear in response to shifting winds

There are a number of different approaches for sea-ice dynamics (by which we mean the horizontal momentum, stress, and transport of the ice) and the applied forcing required to move it. Most large-scale models use the isotropic, viscous-plastic (VP) model of [10] or an extension of it, such as EVP or EAP. The VP model was designed for scales on the order of 100 km. Observations indicate that VP’s continuum and isotropic assumptions do not hold below these scales (in particular, not enough leads are present to make the ice isotropic; [44, 45]). However, when run at high resolution, isotropic, plastic rheologies generate linear kinematic features (LKFs, Fig. 1) such as leads and ridges, which look realistic but are usually not well resolved and may not be oriented correctly [46, 47]. In [48], it is found that many statistical properties are represented very well in a lead-resolving VP model, except for lead intersection angles.

In the consolidated ice pack, recent research has focused on capturing its brittle and/or anisotropic character (e.g., [29, 50–53]) and the landfast ice phenomenon, in which ice becomes grounded and stable in shallow water [54]. Approaches for embedding anisotropy below the grid scale include adding an anisotropic damage model to an existing rheology [29], and abandoning continuum approaches altogether for discrete element models (DEMs, e.g., [55]), which represent collections of ice floes as discrete, Lagrangian elements that interact through contact forces more reflective of the brittle, anisotropic nature of the ice pack. As a transported scalar variable, sea-ice damage could be a key operational diagnostic but needs to be considered in conjunction with concentration changes associated with ice divergence, shear, and compression, in order to determine whether the damage results in greater open water or impassable, ridged ice. Much more research is needed of ice dynamics concerning ice strength, ridging, landfast ice, and interactions with icebergs.

A majority of current operational sea ice models assume constant, neutral transfer coefficients of momentum, heat, and moisture between the air-ice and ice-ocean interfaces. These transfer coefficients, which depend on the large-scale roughness of these interfaces, e.g., from pressure ridges and keels, are expected to vary spatially and temporally. Numerical models [53, 56] suggest the neutral transfer coefficients can vary by as much as a factor of 4 across the pack and a factor of 2 through the year, with impacts on the sea ice mass balance and air-ocean momentum transfer comparable to uncertainties in the sea-ice rheology [53, 57].

Current models also do not account for floe rotation, pitching, rolling, and flexing. In promising work that can span both consolidated and MIZ regimes, some researchers are examining the subgrid-scale interaction and evolution of floes. For continuum models, this takes the form of floe size distributions that evolve in response to freezing, melting, and damage by waves and tides through flexure. Wave energy reaches deeper into the interior Arctic Ocean now than previously [58], which creates ocean mixing in addition to altering the floe size distribution, and thus can affect the heat and moisture flux exchange between the atmosphere and ocean with feedback effects on the ice [41]. DEMs are another approach that may prove useful for capturing the behavior of both regimes. A challenge for DEMs and continuum floe size distribution models is representing the consolidated ice pack, where floes are aggregated into massive ice plates.

Large-scale motion and deformation products derived from remote sensing [59] provide power-law scaling metrics for sea-ice deformation in space and time [60], as well as export fluxes through gateways such as Fram Strait [61]. If Lagrangian drifters were implemented in the models, motion products could be compared more directly. Recent work seeks to analyze and characterize LKFs in the ice based on fracture angles, lengths, densities, growth rates, and lifetime (e.g., [48, 62]).

**Fig. 2:**
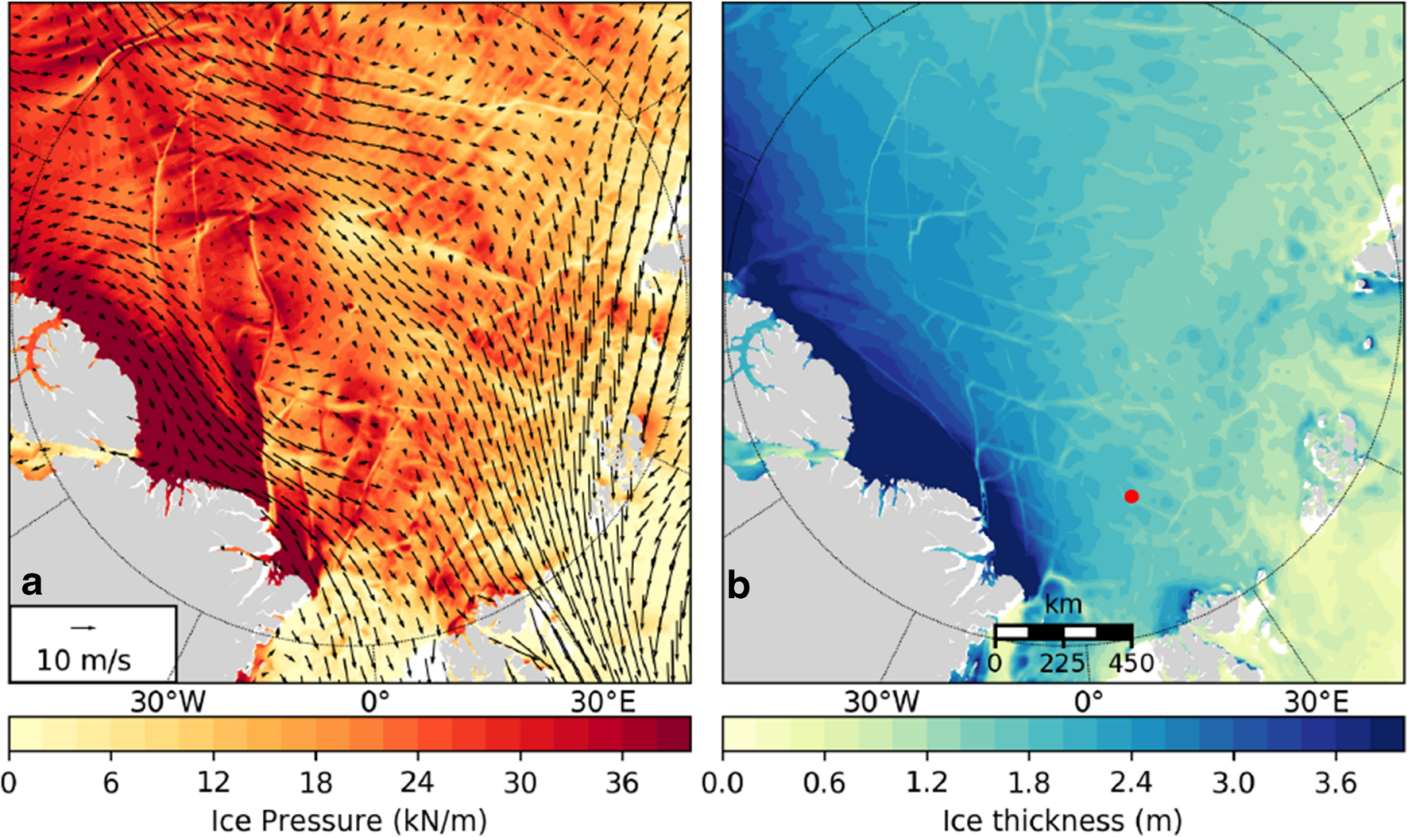
24-h forecast initiated at 00 UTC on 24 April 2020, produced by CCMEP in support of the MOSAiC expedition (the red dot shows the Polarstern position), using the Canadian Arctic Prediction System (CAPS), with NEMO and CICE at 1/12^∘^ grid (4–5 km in the Arctic) coupled with the $\sim 3$ km GEM atmosphere model. **a** Sea-ice pressure (kN/m) and surface winds (m/s). **b** Sea-ice thickness (m). Linear kinematic features appear in response to shifting winds, with high pressure in areas of convergence and thicker ice

Operational forecasts often need information such as sea-ice pressure at subgrid scales, e.g., in the vicinity of vessels (Fig. 2). The Risk Index Outcome (RIO, [63]) metric provides information that helps ship captains decide whether to go and at what speed. However, even 5-km model grid cells may be too large for such applications, and the continuum assumption inherent in many models implies that they are invalid at those scales. This mismatch may be resolved through statistical interpretation of the model output, i.e., probabilistic forecasts.

#### Evaluation and Data Assimilation

Approaches used to objectively evaluate models’ representation of sea-ice characteristics and evolution also depend highly on the application. Seasonal sea-ice prediction skill is significantly affected by the choice of the verification product [63, 64]. For properties above the subgrid scale, remote sensing provides a foundation to compare quantities such as ice concentration, and thickness and freeboard (e.g., [65–67]).

Data assimilation offers a mathematically robust framework for integrating observations in models for many purposes, including state estimation, initialization, and parameter calibration [68]. It can also be applied to optimally design sea-ice monitoring and analysis systems [69, 70].

Short-term forecasts are considered an initial value problem, for which data assimilation provides an optimal initial state. When the lead time of a forecast is increased, the significance of the initial condition decays at varying rates. For example, the “memory” of ice thickness is long, and so ice thickness is sometimes assimilated for seasonal ice prediction (e.g., [32, 71–73]). Meanwhile, assimilating ice velocity is problematic because the initial condition is quickly lost—ice motion is primarily a function of wind at short time scales. Biases in the model state are particularly problematic, and if unaddressed, relaxation to the natural model state can dominate the forecast period. On the other hand, assimilation may render many details of the model physics irrelevant. One disadvantage of data assimilation is that it may change the natural state of the model and create undesired features in a forecast, including significantly altered processes and feedbacks [74].

A primary issue for short-term forecasts based on coupled ocean and sea ice models is bias and uncertainty in the atmospheric forcing, along with a lack of data to properly constrain the models. This is particularly problematic where satellite data is ambiguous, near the coasts, and in the marginal ice zone, areas with numerous marine forecast users.

Ensembles can be used for data assimilation to determine the uncertainty of a model prediction. The ensemble spread represents all uncertainties originating from forcing and physical parameterizations within a modeling tool; missing processes create immense uncertainties in forecasts. For instance, before landfast ice was parameterized in models, [75] had to disable the sea-ice model dynamics to improve the ensemble spread of ice predictions within the Canadian Archipelago.

### Model Complexity

A critical consideration for operational tools is their complexity in terms of maintenance and computational costs, validation of upgrades, and characterization of uncertainty. Greater model complexity is associated with increased human and computational resources and tends to feature larger state vectors and more free parameters across a greater variety of parameterizations. Operational centers require a stable code base, and often prefer simpler codes because of the number, length, and frequency of runs performed each day. Increased complexity is warranted if it improves the atmospheric forecast, or to meet demand for other sea-ice parameters as products. One aim of added complexity is to provide model output that is comparable with observations, in terms of variables’ definitions and their simulated quality of mean sea-ice state and variability.

Another reason to include more detailed sea-ice parameterizations is to better capture the physical processes themselves with more faithful feedback and sensitivity to perturbations [76]. For instance, the same sea-ice processes are at work in the Arctic and Antarctic, but the resulting icepack characteristics and behavior are different because the relative balance and importance of the various processes differ between the two hemispheres. This is largely due to differences in the atmosphere and ocean forcing on the sea ice, but a complete suite of primary sea-ice processes is needed to explore their impacts, with significant implications for predictive performance of the models in the two hemispheres. Although the primary sea-ice processes and their coupled feedbacks are critical to simulating the Earth system, the most effective model upgrades for improving sea-ice predictions might be made in the atmosphere and ocean components of coupled models, or in the coupling mechanisms themselves, which may have a greater impact on short-term forecasts than details of the sea-ice physics.

Both continuum and discrete element models face computational challenges associated with communication in a parallel computing environment and storage of ever-increasing amounts of output data, with clear impacts on the generation of sea-ice diagnostics needed for applications. Physically based statistical analyses and reduced-order modeling can decrease the data volume in a comprehensible way. Machine learning can also be used to train parameterizations within physical models, such as the floe-contact models needed for DEM, and holds great promise for revolutionizing the data-intensive aspects of Earth system prediction.

Model complexity in the form of multiple options for representing the physics adds value for users, who can choose the level of complexity relevant for their problem. For example, simpler configurations with coarse resolution are preferred for long (e.g., millenial) simulations and large ensembles, and simpler sea-ice model configurations might also be appropriate if data assimilation dominates the sea-ice response. While detailed, complex physical descriptions are fundamental for understanding Earth system processes and their interactions, being able to reduce the model to a simpler version enables experimentation, and mechanisms can be prioritized for further modeling and observational study. Moreover, developing a common set of integrated modeling tools entrains capability from the whole community, including research model development and operational validation resources.

## Vision for the Future

The next generation of sea-ice models and parameterizations is being developed while application groups utilize and continue to improve existing models. This approach includes three phases: operational applications using older, well-tested codes; research groups using newer, released versions of codes and updating them with incremental development; and model developers building novel algorithms and modeling frameworks.

A hierarchy of models (or model configurations) is necessary to address the variety of scientific questions spanning operational needs, process understanding, and climate research. A key question is whether to create a simple model to address each scientific problem, or one all-purpose modeling framework that can address a range of problems by selecting relevant parameterizations.

Sea ice is a highly complex material, and implementing numerous options for every process into a single model framework can become unwieldy. Thus, a diversity of model frameworks is useful to better understand alternative mechanisms and balances. The modeling paradigm for coupled, Earth system models has long been modular, allowing components such as the ocean model to be swapped with other options. Now, the components themselves are moving toward mix-and-match subcomponents. For instance, the Discrete Element Model for Sea Ice (DEMSI [77]) uses the column physics from CICE (“Icepack”), with a molecular-dynamics–based model (LAMMPS [78]) underlying the sea-ice dynamics. The ability to combine or link technologies within a common set of frameworks could allow modelers to approach the multiscale, multiphysics challenges of predicting local sea-ice conditions in the context of regional and global change. For example, nested models are commonly used now, in which a large-scale, continuum model provides boundary conditions for an embedded, higher-resolution domain. Combining technologies could enable a DEM “super-parameterization” [79, 80] approach in the embedded domain, or DEM might be applied near the ice edge or coastlines within the continuum model framework, to better capture floe-scale effects at the high resolutions and short time scales needed for navigation.

Although a single model framework is unlikely given the diversity of funding agencies, research interests, and user needs, a common set of metrics and data assimilation tools could be jointly developed and deployed online. Modeling tools necessarily are configured to meet the demands of the problem at hand, but model-agnostic analysis tools that incorporate recommended sea-ice metrics [81], standard operational products from sea-ice models, and a common set of observational datasets would be broadly useful across the sea-ice modeling community, providing a pathway for research and operational centers to take better advantage of existing observations and modeling capabilities as new analysis tools are added. Likewise, data assimilation tools to calibrate model parameters and initialize predictions could be shared if the coding were independent of the central sea-ice model code, a significant challenge. Some tools are becoming available, [e.g., 82, 83].

These types of shared resources foster communication across the modeling and observational communities. To build the modeling, observational, and deployed capabilities that meet society’s needs, dedicated resources are needed to engage the entire community of developers and users in a co-design “value cycle” in which each community contributes and responds to the needs of the others [84, 85]. A key element of this vision is enhanced communication among research communities, operational centers, and stakeholders outside of the scientific realm, such as policymakers.

## Summary

Although the same models can be used for both research and operational applications, in practice this is complicated, with different products and parameters needed, or needed on very different spatial scales. Computational resources present considerable constraints for both research and operations, driving choices of included physics as well as spatial and temporal resolution, simulation length, and ensemble size. Data assimilation is necessary for initializing short-term forecasts, for which sea-ice physics parameterizations that mainly influence longer term feedbacks are likely irrelevant. The main parameters desired by mariners are already available in large-scale sea-ice models, but validation metrics and probabilistic risk information need to be developed. These parameters may not be accurate enough for operational use, requiring additional research and development in new or refined approaches.

A community framework for sea-ice modeling would seek to leverage the whole community for model development, verification, and validation toward a goal of mutually useful, flexible, robust sea-ice modeling tools. With a strong foundation in physics, computer science, and observations, we could use models to make recommendations for targeting observations as well as predicting the sea-ice state and future change. We recommend creating an international benchmarking product for model intercomparisons and forecasts, as an initial, coherent, directed strategy toward a sea-ice modeling and observations co-design cycle valuable for both research and operations.

Shared code is broadly exercised and understood, but a mix of code frameworks is likely to continue. For instance, data assimilation methods are usually tightly intertwined with the physical models, making the code difficult to share. We encourage code sharing when-, where-, and however it makes sense, recognizing that code diversity is also valuable. Within a common conceptual framework, this can take the form of multiple model frameworks (e.g., different dynamical cores) with shared elements (e.g., column physics). Defining the same basic output is crucial, along with a variety of common and unique metrics for applications.

Thus, we need revolution, evolution, and the status quo, simultaneously. Because of limitations in the inherent assumptions of continuum models, short-range forecasting needs DEM, but big changes are not needed for standard climate scales—evolution is sufficient. In particular, VP models continue to be useful because they produce desirable features (anisotropy, tensile stress, scaling laws), although some of the features are not realistic (e.g., the intersection angles of LKFs). We can continue to use continuum models for climate studies, but high-resolution simulations need an alternative. DEM is a promising, revolutionary candidate for the heterogeneous, anisotropic ice pack at fine spatial scales.

## Data Availability

Not applicable.

## Footnotes

1 The title uses the phrase “modeling tools” to avoid the ambiguous term “model,” which can refer to any representation of a given process or system, from an elegant set of mathematical equations to a million lines of numerical code. In this paper, the relevant definition of “model” should be clear from the context in which it is used.
